# Transcriptional control of cuticle formation during development

**DOI:** 10.1093/jxb/eraf521

**Published:** 2025-11-25

**Authors:** Linsan Liu, Chiara Campoli, Sarah M McKim

**Affiliations:** Division of Plant Sciences, University of Dundee, Dundee, UK; The James Hutton Institute, Dundee, UK; Division of Plant Sciences, University of Dundee, Dundee, UK; Iowa State University of Science and Technology, USA

**Keywords:** Cuticle, cutin, fruits, organ development, reproductive strategy, transcription factor, wax, wax bloom

## Abstract

The cuticle is a chemically complex, lipid-rich seal covering aerial tissues in land plants. Secreted by the outer epidermis, cuticles protect against desiccation, organ fusion, pathogen attack and UV radiation exposure. Cuticles also mediate key interactions during reproduction and play other roles depending on the tissue, stage, and species. Reflecting these diverse functions, cuticle composition and structure can vary widely. We review the transcriptional control important for cuticle formation, with special attention to broadly conserved transcription factors where selective regulation of downstream targets drive cuticle specialization. We also discuss how transcriptional control of the cuticle intersects with networks controlling plasticity and cell fate, and explore their translational potential for crop improvement.

## Teaser

Transcriptional regulation controls cuticle formation and cuticle variation across developmental stages, tissues, and species.

## Introduction

Epidermal surfaces of land plants secrete a continuous hydrophobic cuticle that protects the plant body from threats above ground. Most importantly, complex mixtures of mostly hydrophobic polyesters and waxes in the cuticle work together to severely limit water diffusion from internal tissues into the atmosphere. To transpire and exchange photosynthetic gases despite this diffusion barrier, land plants use interspersed cuticle-interrupting epidermal pores, called stomata. Non-stomatal water loss varies widely between species and tissues depending on cuticle thickness, structure, and chemical composition, characteristics which influence other cuticular functions such as repelling pests and pathogens, reflecting UV light, and preventing organ fusion. Cuticles also carry out specialized roles such as attracting pollinators, mediating stigma and pollen interactions, and trapping water. Given these multiple adaptive roles, cuticle formation and elaboration contribute to plant success in different ecological niches and crop performance, especially in changing climates. How plants regulate cuticle formation in different species, tissues, and developmental stages remains a major question; however, transcriptional regulation plays a central role since the cuticle's core metabolic and transport machinery is regulated mostly at the transcriptional level. Here we review transcriptional control of cuticle formation during development, considering both conserved and divergent regulatory modules as well as interactions with epidermal differentiation and external cues.

## Core cuticular machinery and conserved transcriptional activators

**Fig. 1.**
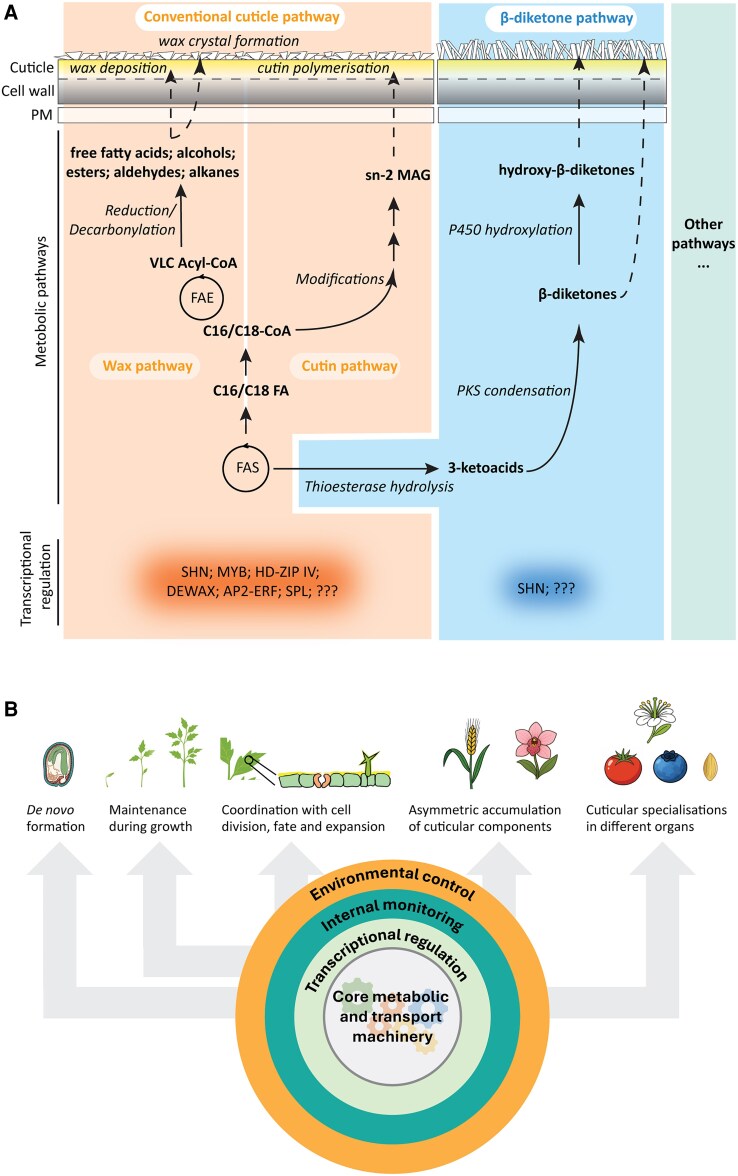
Multiple levels of regulation of cuticle development. (A) Transcriptional and metabolic regulation of cuticle core machinery. In the conventional pathway, a suite of transcription factors regulates the expression of genes encoding the core cuticle metabolic machinery controlling wax and cutin formation. The β-diketone pathway intercepts intermediates from the FAS complexes to produce β-diketones and their derivatives. Other specialized cuticle pathways may be present. (B) Regulatory pathways are influenced by internal signals (e.g. developmental age and cell fate) and environmental cues (e.g. temperature, humidity, and light) so that distinct cuticle components are synthesized, transported, and assembled in a tissue and developmental stage-specific manner. FA, fatty acid; FAS, fatty acid synthase; FAE, fatty acid elongase; sn-MAG, *sn*-monoacylglycerol; PM, plasma membrane; VLC, very long chain. The tomato, blueberry, and flower images were generated using ChatGDP; all other images were made using digital drawing software.

Best understood in leaves, cuticle typically consists of a cell wall associated cutin polyester matrix embedded with phenolics, polysaccharides, and waxes, and covered with epicuticular waxes forming smooth films or rough, often crystalline glaucous structures (Yeats and Rose, 2013; González-Valenzuela *et al*., 2023). The core cuticle machinery carries out biosynthesis, transport, and assembly of cuticle components (Fig. 1A). Cuticular waxes, commonly dominated by ‘ubiquitous’ very long chain fatty acids (VLCFAs, C>20) and their derivatives, are elongated from C16 and C18 precursors by endoplasmic reticulum (ER)-localized multienzyme fatty acid elongases, including 3-ketoacyl-CoA synthases (KCSs) (Lee and Suh, 2022). Cutin synthases (CUSs) oxidize and esterify C16 precursors to make cutin monomers, later polymerized outside the cell (Fich *et al*., 2016). We understand less about the transport of these cuticle components although lipid transport proteins and ABCG transporters are involved (Elejalde-Palmett *et al*., 2021; Philippe *et al.*, 2022). Decades of biochemical and genetic studies on mutants with visible cuticle changes, including *glossy*, *shiny*, and *eceriferum* (*cer*, defined as reduction or absence of wax, Lundqvist and von Wettstein, 1962), mutants showing increased sensitivity to water loss, such as the *wrinkled* mutants, and/or cuticle integrity mutants showing organ fusion, helped define and characterize genes encoding both core cuticle machinery and their transcriptional regulators (Koornneef *et al*., 1989; Samuels *et al*., 2008; Hansson *et al*., 2024). The first regulator identified to control cuticle formation, the Arabidopsis APETALA2 (AP2)-ethylene responsive factor (ERF)-type SHINE (SHN)/WAX-INDUCER (WIN) transcription factor, was identified as a gain-of-function overexpression allele that caused increased wax and cutin load (Aharoni *et al*., 2004; Kannangara *et al*., 2007). We now know that SHNs upregulate genes encoding cuticular machinery across plant species, along with other broadly conserved transcriptional activators including WRINKLED (WRI) ERFs, R2R3 MYELOBLASTOMA (MYB), and class IV homeodomain-Leu zipper (HD-ZIP IV) transcription factors (Park *et al*., 2016; Niklas *et al*., 2017; Kong *et al*., 2020; Xu *et al*., 2021; Kim *et al*., 2022) (Fig. 1A; Table 1). Transcriptional activators within and between families also interact to regulate cuticle formation (Table 1). For instance, Arabidopsis MYB94 and MYB96 directly interact to promote cuticular wax biosynthesis, especially in response to stress and abscisic acid (ABA), a role conserved across species (Lee and Suh, 2022), while regulatory interplay in Arabidopsis between MIXTA-like MYBs (MYB106 and MYB16) and SHN transcription factors promotes cuticular wax, cutin accumulation and conical cell formation in petals (Oshima *et al*., 2013). In fact, many transcriptional regulators important for cuticle formation also influence other epidermal traits (Gray *et al*., 2000; Aharoni *et al*., 2004; Wu *et al*., 2011; Buxdorf *et al*., 2014; Bres *et al*., 2022), and respond to internal and environmental cues, suggesting intersecting and plastic transcriptional networks regulate multiple epidermal traits important for plant performance (Fig. 1B; Table 1). Understanding these dynamics will help explain cuticle formation and show routes for translational impacts in crops.

**Table 1.** Transcriptional modules important for cuticle formation and their interactions

Regulatory modules	Factors by species	Traits	Regulated by and/or interacts with	References
APETALA2 (AP2)-ethylene responsive factors (ERFs)	Arabidopsis: AtSHN1/WIN1, AtSHN2, AtSHN3	Cuticular wax Cutin Floral organ nanoridges Stomatal density and trichome formation Organ nanoridges Likely cell wall	AtMYB106, AtMYB16 Gibberellin signalling AtSHN1/2 directly activated by TCP15	Aharoni *et al*. (2004); Broun *et al*. (2004); Kannangara *et al*. (2007); Shi *et al*. (2011); Oshima *et al*. (2013); Camoirano *et al*. (2020, 2021)
	Tomato: SlSHN3, SlSHN2	Cutin in fruit cuticle	Not yet reported	Oshima *et al*. (2013); Bres *et al*. (2022)
	Rice: OsWR1, OsWR2	Positive regulator of wax synthesis	OSWR2 is transcriptionally repressed by TT2 through a Ca^2+^ signalling cascade in response to temperature	Wang *et al*. (2012); Kan *et al*. (2022)
	Apple: MdSHN3	Cutin in fruit cuticle	Not yet reported	Lashbrooke *et al*. (2015b)
	Cucumber: CsSHN1	Cutin in fruit cuticle	Not yet reported	Rett-Cadman *et al*. (2019)
	Wheat: TaSHN1	Positive regulator of leaf cuticular wax and cutin	Leaf activated by TaMYB74	Bi *et al*. (2016)
	Barley: HvWIN1	β-Diketone wax bloom	Likely downstream of HvYDA1 and HvBRX-Solo epidermal patterning	Liu *et al*. (2022a); McAllister *et al*. (2022)
	Barley: NUD	Grain to hull adhesion	Not yet reported	Taketa *et al*. (2008)
	*Physcomitrium patens*: PpWIN1	Cutin and cuticular wax biosynthesis	Not yet reported	Kim *et al*. (2022)
	Arabidopsis: AtWRI	Cuticular wax in stems	Up-regulated by LEAFY COTYLEDON2	Maeo *et al*. (2009); Park *et al*. (2016)
	*Medicago sativa*: WXP1	Wax load, particularly alcohols	Not yet reported	Zhang *et al*. (2005)
	Arabidopsis: AtDEWAX	Negative regulation of cuticular wax	Interacts with SPL9 and is down-regulated by MYB-SHAQKYF transcription factors, MYS1 and MYS2	Go *et al*. (2014); Cui *et al*. (2016); R.-J. Li *et al.* (2019); Q. Liu *et al.* (2022)
	Arabidopsis: AtDEWAX2	Negative regulation of cuticular wax	Not yet reported	Kim *et al*. (2018)
	Apple: MdDEWAX	Negative regulation of cuticular wax	Not yet reported	Man *et al*. (2024)
	Maize: ZmGLOSSY	Cuticular wax in juvenile tissues	Repressed by miR172	Moose and Sisco (1994); Lauter *et al*. (2005)
MYB	Arabidopsis: MIXTA-like AtMYB106, AtMYB16	Cutin and cuticular wax Petal conical cells Trichomes Cuticular nanoridges	Interacts with and up-regulates AtSHN Induced by ABA AtMYB16 repressed by SPCH AtMYB106 activated by TCP15	Oshima *et al*. (2013); Cui *et al*. (2016); Camoirano *et al*. (2020, 2021); S.-L. Yang *et al.* (2022)
	Orchid: PaMYB9A1, PaMYB9A2	Petal cuticular wax enriched in *n*-alkanes and primary alcohols Conical petal cells	Not yet reported	Lu *et al*. (2022)
	Liverwort: MIXTA-like MpSG94	Cutin Pappilate epidermal cells	Not yet reported	Xu *et al*. (2021)
	Wheat: TaMIXTA1, TaMIXTA2	Leaf cuticular wax	Not yet reported	Wang *et al*. (2024)
	Tomato: SlMIXTA	Cutin Conical epidermal cells	Downstream of SlSHN3	Lashbrooke *et al*. (2015a)
	Arabidopsis: AtMYB96/AtMYB94, AtMYB30	Cuticular wax in stems	Drought induced ABA Ubiquitination of MYB30 and MYB96 by MIEL1;	Seo *et al*. (2011); Marino *et al*. (2013); Lee *et al*. (2014, 2016); Cui *et al*. (2016); Gil *et al*. (2017)
	Arabidopsis: AtMYB74	*De novo* cuticle following organ abscission	ABA Oxidative stress	Ortiz-García *et al*. (2022); Wen *et al*. (2025)
	Wheat: TaMYB74	Leaf cuticular wax and cutin	Drought induced Activates TaSHN1	Bi *et al*. (2016)
	Wheat: TaMYB30		Direct target of TaHY5, regulated by TaHDA19 and TaUVR8 in response to UV-B light	Wang *et al*. (2025)
	Maize: ZmMYB94/FDL1	Embryonic and seedling cuticle	Drought induced	La Rocca *et al*. (2015); Castorina *et al*. (2020); Liu *et al*. (2021)
	Apple: MdMYB94	Leaf cuticular wax, fruit peel cuticle	Not yet reported	Jiang *et al*. (2022); Liu *et al*. (2025)
	Apple: MdMYB30	Leaf cuticular wax Fruit peel cuticle	SUMOylated by MdSIZ1 E3 ligase	Zhang *et al*. (2023)
	Apple: MdMYB62	Negative regulation of leaf cuticular wax	Suppresses MdHDG5	Cao *et al*. (2025)
	Apple: MdMYB106	Leaf cuticular wax	Responsive to nitrogen Targeted for ubiquitination by MdBT2	Jiang *et al*. (2024)
	Peach: PpMYB25, PpMYB96	Fruit cuticular wax Fruit trichome development	Not yet reported	Lu *et al*. (2022)
	Tomato: SlMYB72	Fruit cuticle biosynthesis	Enhanced through interaction with SlTAGL1	Wu *et al*. (2024)
	Tomato: SlMYB31	Leaf and fruit cuticular wax biosynthesis	Interacts directly with WOOLY in the same pathway	Xiong *et al*. (2020)
HD-ZIP IV	Apple: MdHDG5	Leaf cuticular wax	Ubiquitination by MdMEIL1 Suppressed by MdMYB62	Cao *et al*. (2025)
	Maize: ZmOCL1	Cuticle biosynthesis	Repressed by an RDR2-dependent small RNA, small1	Javelle *et al*., (2010); Klein-Cosson *et al*. (2015)
	Rice: ROC4	Wax synthesis	Degraded by the RING-type protein DHS	Wang *et al*. (2018)
	Arabidopsis: AtHDG1	Cuticle biosynthesis	Interacts with AtCFL	Wu *et al*. (2011); Li *et al*. (2016)
	Tomato: SlCD2	Tomato fruit cutin biosynthesis	Forms a regulatory module with SlGRAS9, SlZHD17, SlMBP3, and SlMIXTA-like to control cutin formation; Promoted by SlNOR-like1	Isaacson *et al*. (2009); Nadakuduti *et al*. (2012); G.-S. Liu *et al.* (2024); Shi *et al*. (2025)
WW	Arabidopsis: AtCFL	Negative regulator of cuticle	Antagonistic to HDG1 and directly interacts	Wu *et al*. (2011); Li *et al*. (2016)
NAC	Tomato: SlNOR-like1	Cutin biosynthesis Negative regulation of cuticular wax biosynthesis and transport	Promotes SlCD2 Repressed by SlCNR	G.-S. Liu *et al.* (2024); Chen *et al*. (2025)
bHLH	Arabidopsis: AtZHOUPI	Embryonic cuticle	Interacts with bHLH ICE1	Denay *et al*. (2014)
SPL	Arabidopsis: AtSPL9, AtSPL13	Promote leaf cuticle biosynthesis	Repressed by miR156 SPL9 repressed by DEWAX	R.-J. Li *et al.* (2019); Huang *et al*. (2024)
	Tomato: SlCNR (SBP)	Negative regulation of fruit cuticular wax	Binds to and represses the transcription of SlNOR-like1	Chen *et al*. (2025)

Species as indicated. At, *Arabidopsis thaliana*; Cs, *Cucumis sativus*; Hv, *Hordeum vulgare*; Mp, *Marchantia polymorpha*; Md, *Malus domestica*; Os, *Oryza sativa*; Pa, *Phalaenopsis aphrodite*; Pp, *Physcomitrium patens*; Pp, *Prunus persica*; Sl, *Solanum lycopersicum*; Ta, *Triticum aestivum*.

## Cuticle biogenesis, maintenance, and limitation

Above-ground plant tissues established by primary growth maintain, extend, and elaborate the embryonic cuticular barrier (Fig. 1B). The embryonic cuticle forms via a monitoring system that adjusts gene expression to generate and maintain a continuous barrier (Doll and Ingram, 2022). While embryonic cuticle properties vary from the relatively permeable in Arabidopsis (González-Valenzuela *et al*., 2023) to the thick, impermeable embryonic cuticles in maize, the latter associated with FUSED LEAVES1 (FDL1)/ZmMYB94 up-regulation of a *3-KETOACYL-CoA SYNTHASE* (*KCS*) gene (La Rocca *et al*., 2015; Castorina *et al*., 2020; Liu *et al*., 2021), embryonic cuticle mutants generally show cuticle fusion between embryonic and true leaves, reflecting the key role of embryonic cuticle integrity in embryonic and seedling organ development.

As tissues emerge and grow into the open air, the cuticular barrier incorporates new material to keep pace with underlying epidermal cell layer growth. Transcriptional profiling in developing maize leaves revealed discrete, asymmetric depositions of cuticle components in step with the proximo-distal gradient of cell division to maturation wherein cuticular waxes shifted from shorter to longer chain molecules, followed by deposition of wax esters and finally cutin in fully elongated cells. (Bourgault *et al*., 2020). These trends correlated with expression modules containing different genes from the *MYB*, *SHN*, and core machinery gene families, including individual *KCS* genes whose isoforms target specific chain-length VLCFAs, as well as modules containing PHYTOCHROME light receptors important for a light-sensitive switch from short to long cuticular waxes (Bourgault *et al*., 2020; Qiao *et al*., 2020). Despite compositional transitions, the cuticle remains continuous throughout organ elongation, suggesting a tuned delivery of cuticular components during growth and differentiation (Fig. 1B). How this happens is not entirely clear; however, cuticle and cell wall damage cues generated during growth may be important to trigger surface reinforcement via reactive oxygen species (ROS) and ABA pathways (L’Haridon *et al*., 2011; González-Valenzuela *et al*., 2023; Kim *et al*., 2025, Preprint). Cells transdifferentiate into new epidermis with cuticle after organ abscission in a MYB74-guided process, also express a suite of stress-responsive genes (Wen *et al*., 2025). The exact molecular circuits used by MYB74 to generate a continuous de novo cuticle are unknown, although MYB74 is known to respond to stresses and ABA (Ortiz-García *et al*., 2022), as discussed later in this review, pointing towards a theme of MYB-driven cuticle formation induced by stress signals.

Inadequate production of cuticular components weakens cuticles, but limiting cuticle accumulation is also crucial for cuticle integrity. For instance, some cuticle fusion mutants, including *bodyguard* (*bdg*) *lacerata* (*lcr*), and *fiddlehead* (*fdh*) mutants in Arabidopsis (resulting from impaired alleles encoding an α/β-hydrolase, a cytochrome P450-dependent protein, and a KCS, respectively), as well as gain of function original *SHN* alleles or transgeic overexpression of *SHN* have excess wax and cutin, increased cuticular permeability and cuticle disruption (Lolle *et al*., 1992; Kurdyukov *et al*., 2006b; Voisin *et al*., 2009). Gain-of-function *SHN* alleles also increase ROS production and show exaggerated hypersensitive responses following stress (Sela *et al*., 2013) while *bdg*, *lcr*, and *fdh* mutants have cell wall defects and increased defence-responsive gene expression, consistent with a compensatory response to perceived cell wall damage (Voisin *et al*., 2009), again linking stress signalling with cuticle biogenesis and/or maintenance.

Other factors restrict cuticular component production by directly interfering with transcriptional activators. For instance, overexpression of the CURLY FLAG LEAF (CFL) WW domain-containing proteins in Arabidopsis and rice inhibits cuticle development and causes organ fusion (Wu *et al*., 2011) due to direct inhibition of HDG1-mediated transcription of *BDG*, *KCS*, and *FDH* gene targets (Li *et al*., 2016), a possibly conserved role across CFL proteins (W. Chen *et al.*, 2024; Zhang *et al*., 2025). Protein turnover via ubiquitination also regulates transcriptional activators, as in the cthe Arabidopsis where ubiquitination of MYB96 limits stem wax biosynthesis (Lee *et al*., 2017) and in apple where ubiquination of HDG5 and MYB106 limit leaf cuticular wax (Jiang *et al*., 2024; Cao *et al*., 2025). Antagonism also occurs at the metabolic level, as shown for KCS3 which inhibits the biosynthetic function of KCS6 to limit cuticular wax deposition in Arabidopsis and moss (Huang *et al*., 2023).

Other negative regulators respond to intrinsic and external cues to modulate cuticle formation. For instance, the day–night cycle of wax deposition may reflect the dark induction of DECREASE WAX BIOSYNTHESIS (DEWAX), an AP2-ERF transcription factor that inhibits the cuticle-promoting SQUAMOSA-PROMOTER BINDING-like transcription factor 9 (SPL9), in a pathway downstream of two MYB-SHAQKYF transcription factors, MYS1 and MYS2 (Go *et al*., 2014; R.-J. Li *et al.*, 2019; Q. Liu *et al.*, 2022). SPL13 is another cuticle-promoting SPL which, along with SPL9, is negatively regulated by miR156 (Huang *et al*., 2024). In fact, differences in cuticle features were key to studies on phase change in plants. For instance, juvenile maize leaves produce thinner, more hydrophobic cuticles decorated with more glaucous epicuticular waxes compared with glossier adult leaves (Poethig, 1990). The maize *glossy15* mutant encodes a defective allele of an *AP2* gene (Moose and Sisco, 1994) whose transcript is normally degraded by miR172 to promote the juvenile to adult transition (Lauter *et al*., 2005). The juvenile to adult decrease in epicuticular waxes in maize contrasts with increased wax in older Arabidopsis leaves, highlighting the species-specific nature of phase changes on the cuticle.

## The wax bloom: a phase and species-specific cuticular modification

The adaptive value of phase-specific wax profiles is intriguing to explore, and relevant to crop resilience. For instance, a striking phase-specific cuticular feature in many *Poaceae*, including wheat, barley, and rye, is the white-blue wax bloom—thick layers of self-assembled epicuticular wax crystals appearing on leaf sheaths, leaf blades, and spikelet glumes during the reproductive phase (Tulloch and Hoffman, 1974; Simpson and von Wettstein-Knowles, 1980; Wang *et al*., 2015). Wax blooms form on leaves and stems across land plants (Barthlott *et al*., 1998; Nguyen *et al*., 2024) and on berries and fruits (Arand *et al*., 2021; Middleton *et al*., 2024). Blooms contribute to light reflectance, moisture retention, pathogen defence, and colour perception (Mulroy, 1979; Johnson *et al*., 1983; Richards *et al*., 1986; Middleton *et al*., 2024). In contrast to intracuticular wax mixtures of VLCFAs and their alkane and alcohol derivatives, wax bloom crystals are composed largely of single, often branched compounds, such as α-ketoalcohols in Brassicas and β-diketones in grasses (Meusel *et al*., 2000; Busta and Jetter, 2018). β-diketones in wheat and barley consist mostly of C31 long chains formed from a substrate recruited from fatty acid synthesis. As such, β-diketone biosynthesis competes with other lipid metabolic fluxes, including biosynthesis of the ubiquitous wax compounds, and accordingly is tightly regulated. In wheat, homologous positive (Wax inducer W1 and W2) and negative (Inhibitor of wax Iw1 and Iw2) regulators control β-diketone production (Adamski *et al*., 2013; Wu *et al*., 2013; Zhang *et al*., 2013; Nishijima *et al*., 2014). Genetic analyses of the most abundant *cer* mutations in barley led to the identification of the *Cer-CQU* metabolic gene cluster, homologous to wheat W1, which encodes three enzymes: a type III polyketide synthase, a cytochrome P450, and a hydrolase/carboxylesterase, responsible for the synthesis of β-diketones in barley and wheat (von Wettstein-Knowles and Søgaard, 1980; Hen-Avivi *et al*., 2016; Schneider *et al*., 2016; Sun *et al*., 2023). Remarkably, the barley polyketide synthase accomplishes β-diketone synthesis in a single-step condensation starting from long-chain precursors recruited in the ER and synthesized in the plastid by the hydroxylase/carboxylesterase, distinct from the gradual elongation pathway of other ubiquitous wax compounds (Sun *et al*., 2023). A single point mutation in a gene encoding a α/β hydrolase, orthologous to barley *Cer-Q*, underlies the *glossy.1* mutant in oat (*Avena sativa*) (Kamal *et al*., 2022), suggesting conservation across *Poaceae*.

The wax bloom represents an important breeding target, as glaucousness is associated with higher yield under both irrigated and drought conditions or drought conditions alone depending on the germplasm. Despite its agronomic importance, the only upstream regulatory gene known is the barley *SHN*, *HvWIN1*, which promotes wax bloom on leaf sheaths and glumes, and transcription of *Cer-CQU* and other wax- and cuticle-related genes (McAllister *et al*., 2022). Interestingly, HvWIN1 does not influence levels of VLCFA wax derivatives in the leaf blades (McAllister *et al*., 2022), in contrast to rice OsWIN1 and Arabidopsis AtWIN1 (Aharoni *et al*., 2004; Kannangara *et al*., 2007; Wang *et al*., 2012), suggesting that SHN transcription factors regulate diverse downstream cuticular components across species. In wheat, β-diketone accumulation is strongly inhibited through a post-transcriptional mechanism where the miRNA miRW1, from the *Iw1* locus, degrades the transcript of the WAX1-CARBOXYLESTERASE (W1-COE) from the W1 gene cluster (Huang *et al*., 2017). Additional loci regulate β-diketone accumulation in cultivated and wild wheats (Nishijima *et al*., 2014, 2018; Wang *et al*., 2014; Zhang *et al*., 2015; Li *et al*., 2020, 2021), although their molecular nature remains undetermined. Since wax bloom deposition requires synthesis and export on specific organs at defined times, other transcriptional regulators are likely involved. As the wax bloom may impart increased resistance to multiple stresses, as discussed later in this review, identification of additional factors that regulate wax bloom chemistry and deposition may prove useful to future-proof crops (Johnson *et al*., 1983; Richards *et al*., 1986).

## Transcriptional modification of cuticular metabolism in reproduction

Cuticles also play central roles in reproductive success. For instance, each pollen grain accumulates a rich coat of metabolites, including VLCFAs, synthesized and extruded by degrading endothecium and tapetum in the anther, that confers resistance to dessication and hydration properties for interacting with the stigma (Bai *et al*., 2019; H. Chen *et al.*, 2020; S. Zhang *et al.*, 2021). MYBs in the tapetum up-regulate three distinctive *KCS* genes to make pollen coat VLCFAs (Battat *et al*., 2019; Z. Zhang *et al.*, 2021), another example of regulating specific KCSs underpinning cuticular diversification. The maize silk, an elongated stigma projection, shows dynamic changes in cuticular metabolism and gene expression along its length such that it emerges from the husk with a protective thickened cuticle (K. Chen *et al.*, 2024). In addition, nanoridges on floral organs, associated with increased cutin load andenhanced *CUS* expression, are important for pollinator attraction and/or attachment, and promoted by SHNs, MIXTA-like MYBs, and SPL1/2 regulators (Li-Beisson *et al*., 2009; Shi *et al*., 2011; Antoniou Kourounioti et al., 2013; Hong *et al*., 2017; Zhu *et al*., 2025). These examples illustrate the vital role of cuticle elaboration in reproductive strategies.

## Transcriptional control of fruit cuticles

Cuticle specializations also occur post-fertilization. In particular, both conservation and specialization of regulatory networks contribute to fruit cuticle formation, which has added relevance to crops. Vital food sources for humans and other animals, fruits are maternal pericarp tissues encasing the seed that facilitate seed development and dispersal. Fruit cuticles contribute enormously to fruit development and post-harvest quality, providing resistance to biotic invasion, preventing cracking, and extending shelf life (Martin and Rose, 2014). Generally thicker than the leaf cuticle, and structurally more coherent due to substantially reduced stomatal density or stomatal occlusion from cuticle accumulation (Jeffree, 2006; Garrido *et al*., 2023), the fruit cuticle, like most leaf cuticles, is mainly composed of cutin, waxes, cell wall polysaccharides linked to cutin, and phenolics (Liu *et al*., 2023). Conserved transcriptional activators important in vegetative cuticles also control cuticular deposition in fruit across species; for instance, SHN proteins promote cuticle biosynthesis in tomato, apple, and cucumber fruits (Shi *et al*., 2013; Lashbrooke *et al*., 2015b; Rett-Cadman *et al*., 2019; Bres *et al*., 2022).

We know most about SHNs in tomato fruit due to its substantial and easily isolated fruit cuticle. Tomato fruit pericarp preferentially expresses *SlSHN3* and *SlSHN2*, with defective mutants showing reduced cutin, decreased pathogen resistance, and shorter shelf life. Early identification of SlCD2, a HD-Zip IV that promotes cutin deposition in both leaves and fruits in tomato (Isaacson *et al*., 2009; Nadakuduti *et al*., 2012), led to discovery of other HD-Zip IV cuticle regulators, including maize OCL1 (Javelle *et al*., 2010) and Arabidopsis HDG1 (Wu *et al*., 2011). A recent study revealed that protein-protein interactions of SlGRAS9, SlZHD17, SlMBP3, SlMIXTA-like, and SlCD2 regulate the expression of *SlCYP86A69*, a cutin biosynthesis gene, in both an antagonistic and a synergistic manner (Shi *et al*., 2025). The fruit cuticle dynamically changes in composition, coverage, and properties during fruit development, associated with stage-specific regulation patterns of cuticular deposition (Mintz-Oron *et al*., 2008). For instance, ripening tomato fruits undergo several physiological and metabolic alterations, such as fruit softening and pigment biosynthesis as the cuticle transitions from soft elastic to more rigid viscoelastic regimes, a trajectory associated with phenolic accumulation and interaction with cell wall modification (Benítez *et al*., 2021; Reynoud *et al*., 2022), and aids fruit softening by providing mechanical support and regulating water status (Benítez *et al*., 2021). Several ripening regulators in tomato also control cuticle formation. For instance, SlTAGL1, SlNOR-like1, and MYB72 control pigment accumulation as well as cuticular deposition during the ripening stage by directly targeting cuticle metabolic genes (Giménez *et al*., 2015; G.-S. Liu *et al*., 2024; Wu *et al*., 2024), while another ripening regulator, SlCNR, negatively controls cutin accumulation by directly repressing cutin-related gene expression (Adaskaveg *et al*., 2021; Chen *et al*., 2025). Similarly, defects in ABA-driven ripening programmes resulted in impaired biosynthesis and integrity of the cuticle in ripening orange (Romero and Lafuente, 2020), while exogenous ABA application to ripening sweet cherries and blueberries up-regulated wax gene expression and increased wax coverage (Gutiérrez *et al*., 2021; Yan *et al*., 2024). These studies suggest that a complex regulatory network coordinates cuticular deposition with other ripening events, providing insight about the importance of cuticle formation during fruit maturation in addition to protective and boundary roles.

Fruit cuticles also show diverse chemical specializations. For instance, tomato has a cutin-rich cuticle with little wax, whereas grape and blueberry are wax abundant, with triterpenoids as major components (Yeats *et al*., 2012; Liu *et al*., 2023). Consistent with this, these fruits up-regulate triterpenoid biosynthetic genes (*BAS*, *CYP716A*, and *CER9*) and regulators such as *DEWAX* orthologues and *MYB30/41/61/96* (Dimopoulos *et al*., 2020; M. Zhang *et al.*, 2021; Yan *et al*., 2024). Building on this regulatory theme, blueberry further diversifies its wax profile with β-diketone deposition, correlating with *Cer-CQU* and *WIN1* orthologue expression (Yan *et al*., 2024). By comparison, bayberry (*Myrica pensylvanica*) uses a different wax strategy: an *sn*-2 monoacylglycerol (MAG)-based pathway—possibly repurposing cutin-associated machinery—that produces abundant, fully saturated surface glycerolipids, reaching ∼32% of fruit dry weight (Simpson and Ohlrogge, 2016). Together, highly adapted and lineage-specific regulatory and enzymatic networks appear to govern cuticular deposition in fruits.

Cuticular specializations are also present in dry fruits. In barley grains, the caryopsis (fruit) cuticle becomes sticky during development, causing the outer protective hulls to firmly adhere to the caryopsis pericarp upon contact, in contrast to other cereals such as rice and wheat where hulls are more easily threshed away. An *SHN* gene in barley, called *NUDUM* (*NUD*), was identified almost 20 years ago as necessary for hull adhesion (Taketa *et al*., 2008). Little is known about the downstream mechanism of NUD, although recent transcriptomic studies suggest that it is involved in fatty acid biosynthesis and cell wall modification (Gerasimova *et al*., 2024). We recently identified a GDSL-motif esterase/lipase that controls hull adhesion independent of NUD function (Campoli *et al*., 2024), adding more complexity to the regulatory network. Deletion of *NUD* during barley domestication led to loss of hull adherence, producing ‘naked barley’ suitable for human consumption. Possibly due to tissue-specific subfunctionalization of NUD to the grain, naked barley can be cultivated without significant agronomic drawbacks (Barabaschi *et al*., 2012; Gerasimova *et al*., 2020; Hisano *et al*., 2025), showcasing how altered function of a single transcription factor can modify cuticle features to benefit crop production. However, to effectively target fruit cuticle traits in breeding, a deeper understanding of the regulatory complexity within fruits and across organs is needed due to pleiotropic effects discussed in the next sections.

## Relationships between cuticle deposition/specialization and epidermal differentiation

Made up of mostly pavement cells, the outer epidermis also contains other cell types, normally regularly distributed within the epidermal cell layer, including the guard cells making up the stomatal pore, as well as defensive trichomes or hairs, cells with mineral depositions, and water-catching projections. Although present within the same cell layer, different epidermal cells have different cuticles. For instance, trichomes of Arabidopsis leaves have higher levels of extremely long-chain hydrocarbons compared to pavement cells (Hegebarth *et al*., 2016), while guard cell cuticles facing the stomatal pore surface form outer cuticular ledges (OCLs) (Jeffree, 2006). Such cell-scale specializations suggest tight associations between cuticle metabolism and cell fate are coordinated with other differentiation events to ensure structural integrity and functional efficiency across the epidermis.

At first glance, cuticle biogenesis could simply follow cell fate establishment, yet evidence across species supports a more fluid regulatory interplay between cuticle formation and epidermal cell type, as both cuticle biosynthesis mutants and altered function of cuticular transcriptional regulators such as the MYBs and SHNs, lead to altered epidermal patterning, including density and arrangement of stomata, trichomes, conical cells, and other specialized epidermal cells (Gray *et al*., 2000; Aharoni *et al*., 2004; Kurdyukov *et al*., 2006a; Wu *et al*., 2011; Oshima *et al*., 2013; Buxdorf *et al*., 2014; Lashbrooke *et al*., 2015a; Bres *et al*., 2022; Lu *et al*., 2022; Zhao *et al*., 2024). While the exact crosstalk between cuticular deposition, epidermal development, and transcriptional control is not completely understood, studying pathways important for the arrangement of specialized epidermal cells has helped resolve some overlap with transcriptional activators of cuticular machinery. For instance, stomata in plants are usually separated from each other by at least one pavement cell. The basic helix–loop–helix (bHLH) protein SPEECHLESS (SPCH), which controls the asymmetric cell division (ACD) initiating stomata formation in Arabidopsis and many other plants, represses the cuticle-promoting MYB16 in stomatal precursors, which when overcome through ectopic *MYB16* expression caused thickened cuticles, aberrant ACD, and stomatal clustering, which was subsequently rescued by cutinase expression (S.-L. Yang *et al.*, 2022). These data suggest both that cuticle deposition influences stomatal fate progression and that stomatal identity factors directly modulate cuticular transcriptional activators. In fact, in Arabidopsis, defects in INDUCER OF CBF EXPRESSION 1 (ICE1), another stomatal bHLH factor that interacts with SPCH (Kanaoka *et al*., 2008), resulted in a more permeable seedling cuticle (Denay *et al*., 2014). ICE1 physically interacts with ZHOUPI (ZOU), a bHLH factor involved in the embryonic cuticle-monitoring pathway, whose mutants show not only defective patchy and permeable cuticles but also stomatal clusters on embryonic leaves (Yang *et al*., 2008; Xing *et al*., 2013; Denay *et al*., 2014). Whether cuticle regulation by ICE1 is mediated by ZOU remains unknown. Considering the temporal association between stomatal lineage progression and gradual cuticle strengthening (Tsuwamoto *et al*., 2008; Creff *et al*., 2019), stomatal fate establishment and cuticular deposition may occur in step so that regulation of cuticular deposition by stomatal regulators reinforces cell identity. S.-L. Yang *et al.* (2022) suggested that MYB16-related stomatal clustering may result from altered mechanical properties of th cuticle which disturbs stomatal ACD, yet whether this mechanical effect also underlies the cuticle patchiness-associated stomatal clustering of the embryonic cuticle-monitoring pathway mutants or epidermal patterning alterations of other cuticle mutants, remains unclear.

Trichome differentiation pathways also interact with cuticle deposition. Knockout of *GL1* (*MYB*) and *GL3* (*bHLH*), which control fate specification of trichomes in Arabidopsis, increased leaf cuticle permeability (Larkin *et al*., 1994; Payne *et al*., 2000; Xia *et al*., 2010), while a more recent study showed that the trichome regulator WOOLLY interacts with SlMYB31 to cooperatively control tomato cuticular wax biosynthesis by regulating the expression of *SlCER6* (Yang *et al*., 2011; Xiong *et al*., 2020). Furthermore, mutation of *TCP14* and *TCP15* genes encoding TEOSINTE-BRANCHED/CYCLOIDEA/PCF family transcription factors controlling trichome branching, resulted in more permeable cuticles and down-regulated cuticle genes including *SHN1/2*, with TCP15 directly activating *MYB106* and *SHN1* expression (Camoirano *et al*., 2020, 2021). We recently revealed that the YODA-like MAPKKK pathway that controls fate and patterning of multiple epidermal cell types in barley, including stomata, prickle hair, and silica–cork cells, also controls cuticular deposition (Liu *et al.*, 2022a), shedding light on an upstream coordinating system. Exploring the interactions between epidermal fate and cuticle regulatory factors will be important to understand surfaces in cereals and potential epidermal reprogramming for crop improvement (Uauy *et al*., 2025).

Epidermal cells further specialize cuticular deposition after differentiation. For instance, sculpting stomatal OCLs in Arabidopsis involves increased expression of genes important for cutin assembly, along with a secreted proline-rich protein and GDSL lipase, in guard cells (Kannangara *et al*., 2007; Li *et al*., 2007; Hunt *et al*., 2017; Tang *et al*., 2020). Epicuticular wax filaments on the leaf sheaths and stems of Taiwan oil millet (*Eccoilopus formosanus*) and *Sorghum* appear associated with specific cell types (Jenks *et al*., 1994; Chemelewski *et al*., 2023; Anggarani *et al*., 2024), aligning with our ideas about epidermal cell identity and wax bloom deposition (Liu *et al.*, 2022a). Gene regulatory network analyses further suggested that the wax deposition in sorghum stems is associated with SbSHN1 and several MYBs (Chemelewski *et al*., 2023). The regulatory relationship between cell type and cuticle features is perhaps not surprising given the primary adaptive role of these cuticular specializations to cell type function. For instance, the stomatal OCL probably prevents water loss by sealing the pore when closed and blocking water entry when open (Schönherr and Ziegler, 1975; Zeiger *et al*., 1987). Future research into the regulation of specialized cuticle metabolism across different cell types will deepen our understanding of epidermal adaptation strategies.

In summary, cuticular deposition appears precisely coordinated with epidermal differentiation and can be further specialized in mature cells. Throughout these processes, cuticle metabolism is likely regulated in a cell type- and developmental stage-specific manner, and closely linked to cell fate and responsive systems, reflecting the broader regulatory networks governing cell development and function.

## Cuticle formation and responses to environmental cues (an additional layer of control)

Transcriptional changes are involved in altered cuticle formation in response to environmental cues (Fig. 1B;  Shepherd and Wynne Griffiths, 2006; Lewandowska *et al*., 2020; Liu *et al*., 2022b; Wang and Chang, 2022). Given the role of cuticles in preventing water loss, response to drought is most studied. Water deficit caused increased wax and cutin levels on leaves of Arabidopsis (Kosma *et al*., 2009), while drought exposure caused wax increases in many dicot and cereal crops (Jefferson *et al*., 1989; Bondada *et al*., 1996; Islam *et al*., 2009; Bi *et al*., 2017; L. Li *et al.*, 2019; M. Chen *et al.*, 2020; F. Yang *et al.*, 2022). MYB transcription factors have a central and conserved role in regulating wax and cutin biosynthesis in response to drought stress. For instance, Arabidopsis and maize AtMYB96/94 and ZmFDL1/MYB94, respectively, up-regulate cuticular wax biosynthetic genes in response to drought (Seo *et al*., 2011; Lee *et al*., 2016; Castorina *et al*., 2020). In wheat, drought-induced TaMYB74 directly up-regulates wax biosynthetic genes *TaATT1* and *TaKCS1* and the activator *TaSHN1* (Bi *et al*., 2016). *SHN* overexpressors in Arabidopsis show enhanced drought tolerance, despite increased cuticular permeability, potentially due to altered cuticle structure, increased wax load and changes in stomatal index (Aharoni *et al*., 2004). Similarly, the overexpression of *TaSHN1* and *SlSHN1* alters cuticular properties and improves drought tolerance in wheat and tomato, while heterologous expression of *SHN* genes in other species also alters leaf cuticular properties and improves resistance to multiple stresses (Al-Abdallat *et al*., 2014; Bi *et al*., 2018; Zhang *et al*., 2019; Djemal and Khoudi, 2021), indicating a conserved role for SHN transcription factors in stress responses. Overexpression of the AP2 transcription factor WXP1 (Wax Production 1) in *Medicago sativa* increased leaf wax crystal density and wax load, particularly of alcohols, resulting in enhanced drought tolerance (Zhang *et al*., 2005). Conversely, the overexpression in rice of the *DHS* (*DROUGHT HYPERSENSITIVE*) gene, which encodes a RING (Really Interesting New Gene)-type protein with E3 ligase activity that degrades the wax-promoting ROC4 HD-ZIP results in less wax and more drought-sensitive plants (Wang *et al*., 2018). In rice, the *TT2* (*Thermotolerance2*) locus, encoding a Gγ subunit of a G protein complex, modulates the Ca^2+^ signalling cascade to reduce wax by transcriptional repression of *OsWR2* following high temperature; however, a natural mutation present in *TT2* of many subtropical and tropical *japonica* varieties leads to a less severe reduction in leaf wax and increased thermotolerance without affecting yield (Kan *et al*., 2022). Interestingly, the wax bloom also shows plasticity, with older Canadian bread wheat varieties showing a greater increase in leaf β-diketones in response to drought and higher yield compared with modern varieties, suggesting potential benefical alleles in older germplasm possibly lost through elite breeding (Kuruparan *et al*., 2024).

Increased light exposure promotes cuticular wax deposition in both monocots and dicots, increasing foliar reflectance without changing cuticle residual transpiration (Herzig *et al*., 2025). In wheat, the wax synthesis regulator TaMYB30 is a direct target of transcription factor ELONGATED HYPOCOTYL5 (TaHY5). TaHDA19 suppresses *TaMYB30* expression by reducing the enrichment of TaHY5 at the promoter region, while UV RESISTANCE LOCUS 8 (TaUVR8) induces *TaHY5*, therefore promoting wax synthesis, in response to UV-B radiation (Wang *et al*., 2025). Cuticles also respond to soil micronutrient availability. Manganese starvation of barley plants caused a 40% reduction in extractable leaf wax associated with increased leaf transpiration (Hebbern *et al*., 2009). Intriguingly, these plants show an increase in trichome and stomatal densities, a thinner cuticle, and changes in epidermal cell size, suggesting that mineral deficiency alters a suite of traits, whose origin may be mechanistically related (Arsic *et al*., 2022). Conversely, mangrove (*Bruguiera gymnorhiza*) exposed to an increased copper concentration showed reduced leaf growth and stomatal closure, and increased leaf wax load, with an increased proportion of primary alcohols and alkanes associated with wax and cutin biosynthetic gene expression (Shang *et al*., 2024), although the factors involved in this transcriptional regulation are currently unknown.

Taken together, multiple environmental cues, including water availability, light quality and intensity, as well as soil geochemistry, influence cuticle formation through post-transcriptional and transcriptional control, often working through MYB and SHNs. That said, engineering thicker or more stress-resistant cuticles can face hurdles in terms of the approaches used as transgenic gene editing or overexpression can present regulatory challenges as well as potential secondary effects on epidermal features and cellular metabolism. For instance, investments in cuticle production could lead to changes in energy flux available for yield (Wang and Chang, 2022) as well as fluxes through lipid biosynthetic pathways, especially those involved in stress responses, such as in maize where increased flux into VLFCA pathways leads to reduced production of jasmonic acid, a critical defense phytohormone (J. Liu *et al.*, 2024). In addition, cuticle permeability may influence stomatal opening through effects on transpiration rate (Monda *et al*., 2021), suggesting functional trade-offs in leaf performance. Due to their highly dynamic response to environmental changes, their role in protection from the environment, and impacts on overall biology, the understanding of cuticle dynamics and the characterization of the genes that modulate cuticle synthesis, export, and deposition in response to stress will be important; it is equally important to measure and anticipate these secondary effects, in order to develop more resilient crops.

## Cuticle evolution and selection

While homologous transcriptional activators promote cuticle formation in multiple species, exact cuticular gene targets and impacts on cuticular chemistry can differ, such as in the aforementioned SHNs which promote VLCFAs in Arabidopsis leaf cuticles and β-diketones in cereal wax blooms (McAllister *et al*., 2022). Comparative studies across the green plant lineage suggest that cuticles became increasingly impermeable as plants colonized more arid terrestrial environments, in step with elaboration and co-expression of gene families encoding core machinery and their transcriptional activators (Niklas *et al*., 2017; Kong *et al*., 2020; Xu *et al*., 2021). For instance, a MYB in the liverwort *Marchantia polymorpha*, MpSBG9, orthologous to MIXTA proteins in vascular plants, promotes cutin but not cuticular wax biosynthesis, suggesting that gain of this latter ability in vascular plants (Xu *et al*., 2021). Interestingly MpSBG9 also promotes conical shape in epidermal cells (Xu *et al*., 2021), suggestive of a deeply ancestral role for transcriptional regulators linking epidermal fate with cuticular features. As evident in this review, cuticle variation in different plant tissues and/or between species usually reflects differences in transcriptional regulation of the core machinery (Busta *et al*., 2021; Petit *et al*., 2021; González-Valenzuela *et al*., 2023; Jolliffe *et al*., 2023), which can include targeting different members of the core cuticular gene family such as the MYB regulation of KCSs involved in pollen coat VLCFAs (Battat *et al*., 2019; Z. Zhang *et al.*, 2021), and/or activating novel enzymatic players such as the case in sorghum where a steroid synthesis gene was recruited to enrich leaf cuticular wax in triterpenoids to increase resiliency (Busta *et al*., 2021). Metabolic specialization and transcriptional regulation may also be regulated by genomic rearrangements: for instance, genes responsible for the distinctive β-diketones biosynthetic pathway are genetically clustered in barley and wheat (Hen-Avivi *et al*., 2016; Schneider *et al*., 2016), which could help coordinate their regulation.

Regulatory differences can also underly variation in the field. For instance, altered cuticular composition conferring better cuticular water retention in alpine compared with foothill ecotypes of *Arabidopsis arenosa* is linked with changes in upstream regulatory motifs controlling expression of core cuticular machinery (Bertel *et al*., 2025). Variation in genes involved in cuticular metabolism and transcriptional regulation may also explain differences in epicuticular wax composition and cuticular water loss in leaves across maize accessions (Lin *et al*., 2022, 2024), while increased expression of a single *KCS* gene may be crucial to synthesize distinctive alkenes in poplar accessions (Gonzales-Vigil *et al*., 2017). Moreover, variation in the apple *HDG5* promoter led to differences in its regulation by an upstream MYB, underpinning changes in apple fruit cuticular wax (Cao *et al*., 2025). Taken together, tweaking transcriptional control of the cuticular machinery probably refines adaptive cuticular properties in both cultivated and natural populations, and represents a possible route to cuticle modification which may avoid excessive pleotropic effects from loss- and gain-of-function approaches.

## Conclusion

The cuticle is essential for plants to survive on land by restricting water loss through the exposed plant surface. Cuticles also fulfil a myriad of other roles, from protecting against other terrestrial threats, mediating favourable interactions during reproduction, and conferring advantageous features on fruits and seed (Fig. 1B). Thus, selecting and/or engineering epidermal variation are attractive prospects to stabilize and improve crop yield under changing climates, and represents an important breeding target (Jolliffe *et al*., 2023). The cuticular machinery is primarily regulated at the transcriptional level exploiting transcriptional regulators is a promising route to modify cuticular features. We show how conserved regulators, especially the SHN and MYB transcription factors, work in multiple species and tissues during cuticle development and specialisations (Fig. 1). While the relationship between transcriptional regulators and cuticle deposition is slowly resolving, we know less about cuticle maintenance during organ development, the interplay between cuticular and cell fate regulators, as well as cuticle plasticity. Newer technologies including single-cell approaches as well as precise spatial chemical profiling, together with classic genetic methods and field experiments, will help elucidate some of these unknowns.
